# Retained Placenta Accreta Mimicking Choriocarcinoma

**DOI:** 10.1155/2015/167986

**Published:** 2015-10-01

**Authors:** Maureen P. Kohi, Gabrielle A. Rizzuto, Nicholas Fidelman, Jennifer Lucero, Mari-Paule Thiet

**Affiliations:** ^1^Department of Radiology and Biomedical Imaging, University of California, San Francisco, CA 94143, USA; ^2^Department of Pathology and Laboratory Medicine, University of California, San Francisco, CA 94143, USA; ^3^Department of Anesthesia and Perioperative Care, University of California, San Francisco, CA 94143, USA; ^4^Department of Obstetrics, Gynecology and Reproductive Sciences, University of California, San Francisco, CA 94143, USA

## Abstract

This case demonstrates a rare event of retained invasive placenta masquerading as choriocarcinoma. The patient presented with heavy vaginal bleeding following vaginal delivery complicated by retained products of conception. Ultrasound and computed tomography demonstrated a vascular endometrial mass, invading the uterine wall and raising suspicion for choriocarcinoma. Hysterectomy revealed retained invasive placenta.

## 1. Introduction

Abnormally invasive placenta is a life-threatening condition that occurs when chorionic villi adhere to the uterine myometrium without normal intervening decidua basalis [1]. The most common complication of invasive placenta is postpartum hemorrhage (PPH), which often requires hysterectomy [2].

Retained products of conception (RPOC) or placental fragments are a common cause of PPH with an incidence of 3%–5% after routine vaginal delivery [3]. Ultrasound (US) is the primary modality during the antepartum period and the modality of choice to evaluate for PPH [4]. Combined gray-scale and color Doppler US allow real-time assessment of the uterine cavity and blood flow, which aid in the diagnosis of RPOC. Early diagnosis is critical for directing clinical management and for preventing associated immediate complications, such as infection, as well as future obstetric complications [5].

Herein, we present a case of retained invasive placenta, which was undetected in the antepartum and mimicked choriocarcinoma in the postpartum period, ultimately resulting in hysterectomy.

## 2. Case Report

A 39-year-old gravida 3 nulliparous female with a dichorionic diamniotic twin pregnancy at 36 weeks and 4 days was admitted to our institution for induction of labor secondary to intrauterine growth restriction (IUGR) of both infants. Her obstetrical history was significant for two prior dilatation and curettage (D&C) procedures. Her antenatal ultrasounds demonstrated two normal placentas without evidence of previa or placental invasion.

She progressed to spontaneous rupture of membranes 18 hours following administration of oxytocin and placement of a transcervical Foley balloon. Once US confirmed vertex lie of both infants, the patient was moved to the operating room (OR) where labor progressed normally with delivery of two female infants weighting 2085 g (Apgar scores of 8 and 8 at 1 and 5 minutes) and 1945 g (Apgar scores of 4 and 8 at 1 and 5 minutes), respectively.

The third stage of labor was complicated by retained placenta, which was extracted manually and with banjo curettage under ultrasound guidance. At the end of the procedure, a thin endometrium was confirmed by US.

Initially, the postpartum course was uncomplicated, and the patient was discharged on postpartum day two in stable condition. However, on postpartum day five, while visiting her infants in the hospital, the patient passed an orange-sized blood clot. A repeat US demonstrated a thin endometrial stripe. For the next several weeks, light bleeding continued.

At the routine 6-week postpartum visit, the patient again passed a large blood clot and her uterus was palpable 2 cm below the umbilicus. At that time, serum quantitative human chorionic gonadotropin (hCG) level was 203 IU/L, and hematocrit was 33%.

**Figure 1:**
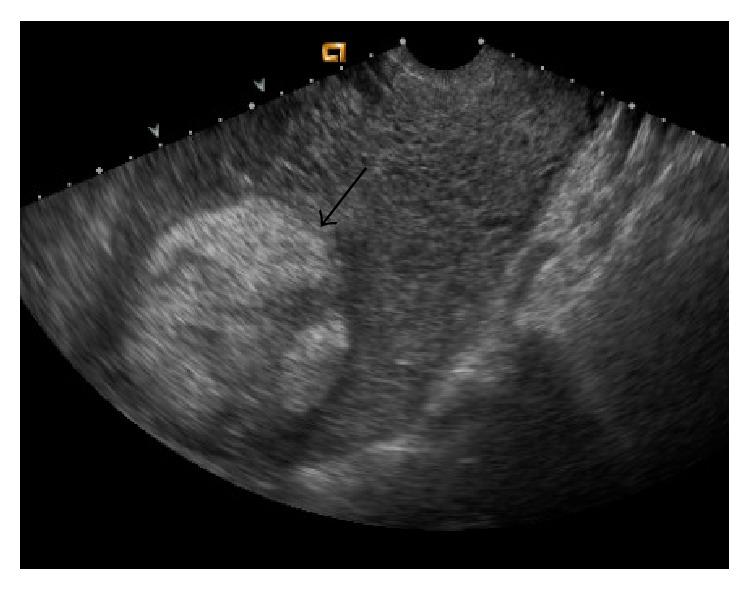
Gray-scale US demonstrates an echogenic mass in the endometrial cavity (black arrow).

**Figure 2:**
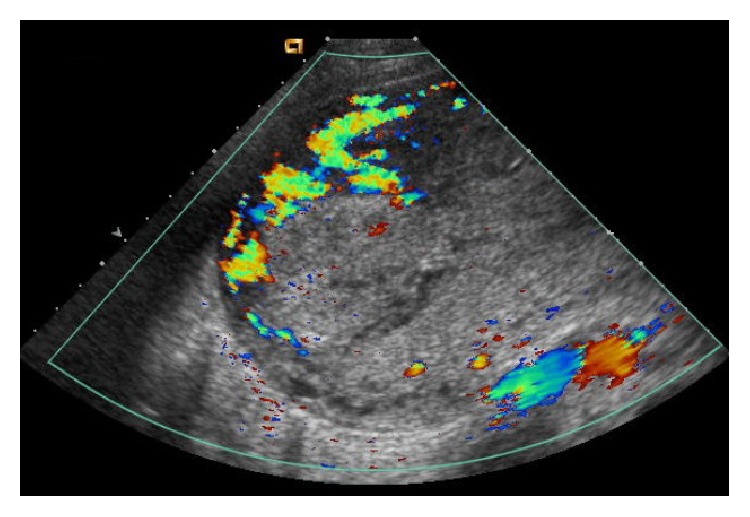
Color Doppler US image demonstrates vascularity in the echogenic mass with extensive vascularity surrounding the mass.

**Figure 3:**
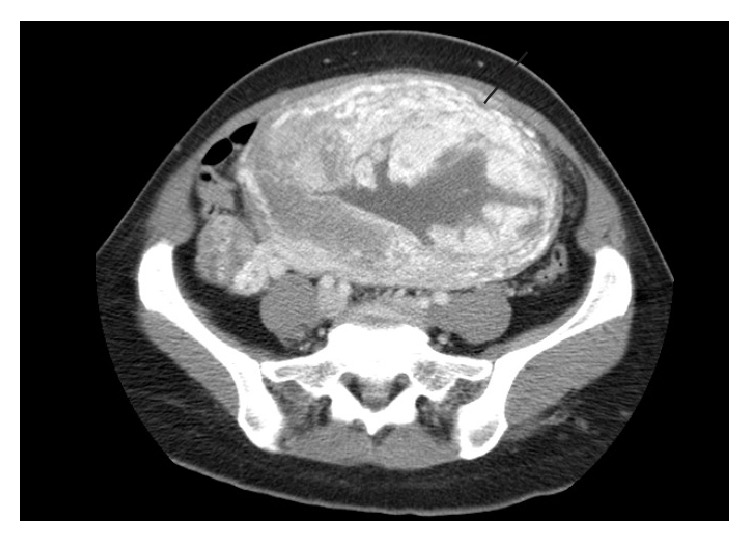
Contrast-enhanced CT image demonstrates hypervascular uterine mass. Note loss of plane between the mass and the uterine wall (white arrow).

**Figure 4:**
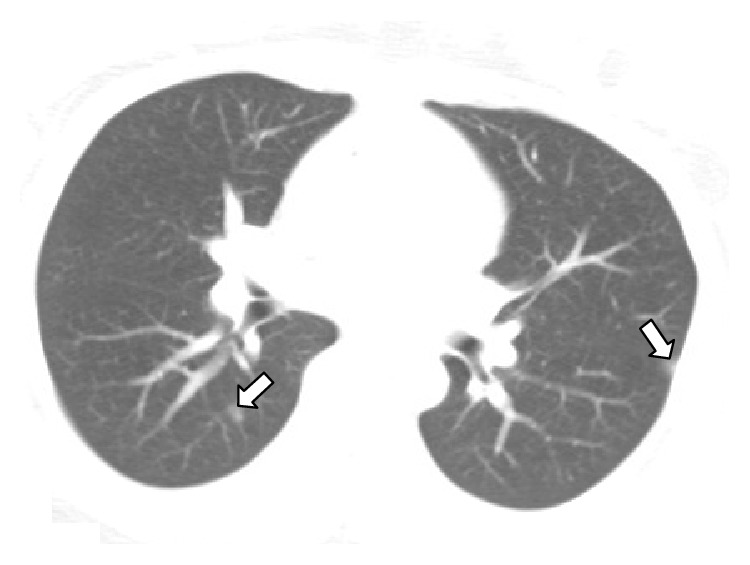
CT image of the chest demonstrates ground glass opacities in the lungs (open arrows).

Transvaginal US showed a large echogenic mass within the endometrial cavity, measuring 9.4 × 8.5 × 6.7 cm (Figure 1). Color Doppler US demonstrated vascularity, predominately in the periphery of the mass (Figure 2). Computed tomography (CT) demonstrated a large hypervascular and heterogeneously enhancing uterine mass measuring 10.4 × 15 × 16.8 cm with diffuse myometrial invasion (Figure 3) and CT chest demonstrated bilateral ground glass nodules (Figure 4). Differential diagnoses included gestational trophoblastic disease versus RPOC, but choriocarcinoma was favored given the hypervascularity noted on CT, the degree of uterine invasion, and the presence of pulmonary nodules, which is worrisome for metastases. RPOC was considered less likely in light of manual and instrumental placental extractions and thin stripe noted US performed in the OR.

Management options included transcervical biopsy for diagnosis (D&C) with frozen section with subsequent hysterectomy in case of malignancy or outright hysterectomy. The patient and her husband did not desire future fertility and preferred hysterectomy.

**Figure 5:**
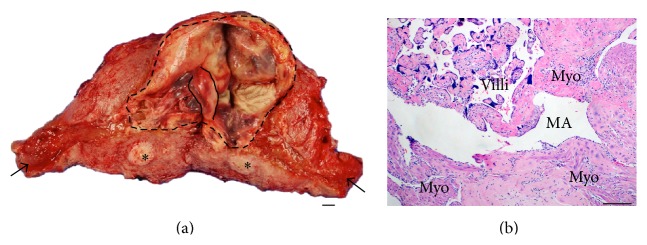
Grossly retained placenta with microscopic evidence of placenta accreta. (a) Gross photograph of hysterectomy specimen bisected in coronal plane shows ~12 × 11 × 3 cm fundal placenta (outlined in dashed lines) with ~3.5 cm umbilical cord (outlined in solid lines) (arrows at cervical os, *∗* = leiomyoma). (b) Hematoxylin and eosin stained microscopic section demonstrates degenerating placental villous parenchyma [villi] adjacent to large bands of myometrial smooth muscle [myo] without intervening decidua [MA = maternal myometrial artery]. Scale bars: (a) 1 cm and (b) 100 microns. Note: due to extensive tissue degeneration at the placenta/myometrial interface, the depth of accreta could not be accurately determined on pathologic examination.

The patient underwent total abdominal hysterectomy, and pathology demonstrated placenta accreta (Figures 5(a) and 5(b)). The postoperative course was uncomplicated, and the patient was discharged on postoperative day four.

## 3. Discussion

Invasive placenta is a condition caused by placental invasion into the uterine wall. Three distinct types of invasive placenta exist, based on the degree of placental villi invasion into the myometrium: placenta accreta (superficial invasion of the basalis layer), placenta increta (deeper invasion of the myometrium), and placenta percreta (deeper invasion involving the serosa and other surrounding organs such as the bladder) [6]. Risk factors for invasive placentation include placenta previa, previous history of cesarean delivery, advanced maternal age, previous uterine surgery, and multiparity [7]. The incidence of invasive placentation has increased fivefold over the past few decades from 1 in 2510 in the 1980s to currently about 1 in 533 pregnancies [7]. The major contributing factor to this is likely the increase in the rate of cesarean delivery and uterine instrumentation [8].

Antenatal diagnosis of invasive placentation is critical and has been shown to decrease maternal morbidity [9]. US is the primary imaging modality for the diagnosis of invasive placenta in the antepartum period [10] with sensitivity of 91% and specificity of 97% [11]. Findings suggestive of invasive placentation on US include intraplacental lacunar spaces, lack of normal retroplacental clear zone, irregularity and attenuation of the uterine-bladder interface, retroplacental myometrial thickness, and bridging vessels between the placenta and bladder wall when using color Doppler [12].

RPOC refers to intrauterine tissue that persists after delivery or termination of pregnancy and is often of placental trophoblastic origin and a common cause of PPH [5]. US is the primary modality for the diagnosis of RPOC. On gray-scale US, the presence of a thickened endometrial echo complex (EEC) of at least 10 mm has a diagnostic sensitivity of 80% [13] and presence of an endometrial or intrauterine mass has a diagnostic sensitivity of 79% [14]. Additionally, color Doppler US can further enhance the diagnosis of RPOC as any vascularity detected in a thickened EEC or mass increases the likelihood of RPOC [5].

CT or magnetic resonance imaging (MRI) can serve as diagnostic adjuncts in complicated cases. However, there is variability of postcontrast enhancement on CT imaging and of T1- and T2-weighted signal intensity depending on the degree of hemorrhage and tissue necrosis [14, 15].

Gestational trophoblastic disease is a rare complication of pregnancy encompassing a group of interrelated diseases ranging from premalignant partial and complete hydatidiform mole to malignant diseases of an invasive mole, choriocarcinoma, and rare placental-site trophoblastic tumor and epithelioid trophoblastic tumor [16]. The serum and urine hCG levels are elevated in this disease process. Choriocarcinoma is a rare trophoblastic tumor characterized by myometrial and vascular invasion with high incidence of pulmonary metastasis in the form of nodules with surrounding ground glass opacities [17].

In the present case, the vascularity of the uterine mass in addition to the myometrial invasion suggested a malignant process as opposed to invasive placenta. In addition, the small ground glass pulmonary nodules in the setting of the invasive uterine mass suggested the diagnosis of choriocarcinoma.

Prior reports have described that retained invasive placenta may mimic acquired arteriovenous malformation [18], leiomyomata [19], and endometrial cancer [20]. Therefore, it is important to consider the diagnosis of retained invasive placenta in patients presenting with PPH who have risk factors for invasive placentation. This is particularly critical prior to performing a D&C for presumed RPOC, which in cases of retained invasive placenta may result in massive bleeding, necessitating an emergent hysterectomy.
